# Comparison of Frozen Embryo Transfer Outcomes Between Uterine Infusion of Granulocyte Colony-Stimulating Factor and Growth Hormone Application in Patients With Thin Endometrium: A Retrospective Study

**DOI:** 10.3389/fendo.2021.725202

**Published:** 2021-12-28

**Authors:** Lei Jiang, Xin Xu, Ziyu Cao, Ni Yang, Shaoqing Wang, Luning Wang, Xiuhua Xu, Qian Li, Baojun Shi, Guimin Hao

**Affiliations:** ^1^ Department of Reproductive Medicine, Second Hospital of Hebei Medical University, Shijiazhuang, China; ^2^ Cardiovascular Platform, Institute of Health and Disease, Hebei Medical University, Shijiazhuang, China

**Keywords:** thin endometrium, frozen-thaw embryo transfer, colony cell stimulating factor, growth hormone, pregnancy outcome

## Abstract

**Objective:**

To investigate the effect of two treatments on the outcome of freeze-thaw embryo transfer for pregnancy assistance in thin endometrium.

**Methods:**

A retrospective study was conducted on 66 patients who failed in the first cycle treated in the reproductive medicine center of the Second Hospital of Hebei Medical University from January 2018 to December 2019. Granulocyte colony stimulating factor (G-CSF) was used through cavity infusion in one group (n=25, and growth hormone (GH) was subcutaneously injected in the group (n=41). The clinical data of the two groups were compared, including morphology and thickness of the endometrium, biochemical pregnancy rate, clinical pregnancy rate, implantation rate, miscarriage rate, and live birth rate in each period of the hormone replacement cycle.

**Results:**

There was no significant difference in age, BMI, AMH, FSH, LH, E_2_, infertility years, number of transferred embryos, basal endometrium, and thickness of endometrium on the day of P administration before and after treatment (*P*> 0.05). After treatment, compared to the GH group, the G-CSF group presented higher biochemical pregnancy rate (56% versus 48.8%; *P*=0.569), clinical pregnancy rate (52% versus 46.3%; *P*=0.655), implantation rate (34.8% versus 27.5%; *P*=0.391), and live birth rate (40% versus 31.7%; *P*=0.493), but the differences were not statistically significant (*P* > 0.05). On the 5th day of treatment, the endometrial thickness in the G-CSF group was thinner than that in the GH group (4.83 ± 0.85 versus 5.75 ± 1.27; *P*< 0.05), but it had no correlation with pregnancy outcome (*P* > 0.05). There was no significant difference in endometrial thickness between the two groups on the 7th, 9th day of treatment and the day of P administration (*P* > 0.05). On the 5th day of treatment, the proportion of endometrial type A morphology in the GH group was significantly higher than that in the G-CSF group (*P* < 0.05), while the type B morphology in the G-CSF group was significantly higher than that in the GH group (*P*< 0.05).

**Conclusion:**

Although G-CSF and GH may not have a role in increasing endometrium, both of them can improve the pregnancy outcomes of patients with thin endometrium in the FET cycle. And the effects of the two treatments were similar.

## Introduction

With the development of vitrification freezing technology, the implantation and live birth rates of FET has met or exceed those of fresh embryo transfer, especially for infertile women with slow embryo development or early elevated progesterone. Hence more and more fertility centers in China and abroad have adopted the whole embryo freezing strategy as a standard treatment option in recent years. Endometrial receptivity, embryo quality, and synchronization of both during the FET cycle are important factors for successful embryo implantation. It was found that endometrial factors account for about 60% and embryonic factors for 40% for pregnancy success rate (1). At present, embryo freezing and thawing technology is more mature and the recovery rate can reach more than 90%, but the clinical pregnancy rate of the FET cycle still fluctuates between 30% to 60% in various fertility centers in China and abroad (2). Therefore, improving endometrial receptivity has become an urgent challenge in the field of reproduction. One of the main manifestations of poor endometrial receptivity is thin endometrium, and most scholars believe that a thin endometrium could be diagnosed with an endometrial thickness <7 mm on the IVF/ICSI trigger day or luteal support day (3, 4). A more satisfactory pregnancy outcome is achieved when the endometrial thickness is at least ≥7 mm or even 9 mm, while the clinical pregnancy rate is lower when the endometrial thickness is <6 mm (5). The treatment of thin endometrium is still being explored, and the current treatments include high-dose estrogen replacement therapy, gonadotropin-releasing hormone agonists, GH, aspirin, sildenafil, cervical curettage, and uterine perfusion. Among them, G-CSF is a glycoprotein that acts mainly on neutrophils to promote their value-added, differentiation and activation. In the field of reproduction, G-CSF has been found to be involved in follicular growth and development, ovulation and pregnancy, and has a bidirectional regulatory role in the maternal-embryonic exchange. A basic experiment shows that G-CSF plays an important role in improving ischemia/reperfusion (I/R) injury of specific tissues (6). GH is a peptide hormone secreted by the anterior pituitary gland and plays an important role in cell growth and metabolism. GH and its receptor GHR and related growth factor (IGF-1) act on human endometrial cells to promote endometrial cell proliferation and endometrial interstitial vascularization and up-regulate the expression of receptivity-related factors (7). The aim of current study was to compare the efficacy of G-CSF and GH on thin endometrium in order to select a more optimal approach for clinical treatment of thin endometrium patients with FET-assisted pregnancy, improving their endometrial receptivity and pregnancy outcomes.

## Materials and Methods

### 1 Data and Methods

#### 1.1 Study Population

The data of patients who underwent the proposed FET cycle at the Department of Reproductive Medicine, Second Hospital of Hebei Medical University between January 2018 and December 2019 were selected for analysis. The ethical approval number is 2021-P037.

The inclusion criteria included: ① age between 25-40; ② fixed GnRH antagonist protocol when control ovarian stimulation, and failed in their first fresh ET;③ conventional hormone replacement cycle protocol in preparation for the second cycle of FET, multiple ultrasound detection of endometrium ≤6 mm (3, 5); ④ at least one 3d embryo frozen, and planned transfer of 1-2 3d high-quality embryos in this cycle; ⑤ IVF/ICSI-ET due to female tubal factors or male factors.

The exclusion criteria included: ① patients with abnormal liver and kidney function, abnormal thyroid function, immune disorders, or hematological disorders; ② patients with repeated implantation failure; ③ patients with combined fibroids, endometrial polyps, endometriosis, or uterine malformations; ④ patients with a hysteroscopic examination of uterine adhesions or endometrial lesions;⑤ patients with a body mass index ≥ 30 kg/m^2^; ⑥ chromosomal abnormalities in couples. This study had been discussed and approved by the medical ethics committee, and all enrolled patients signed the informed consent form with full knowledge.

#### 1.2 Methods

##### 1.2.1 Grouping

**Figure 1:**
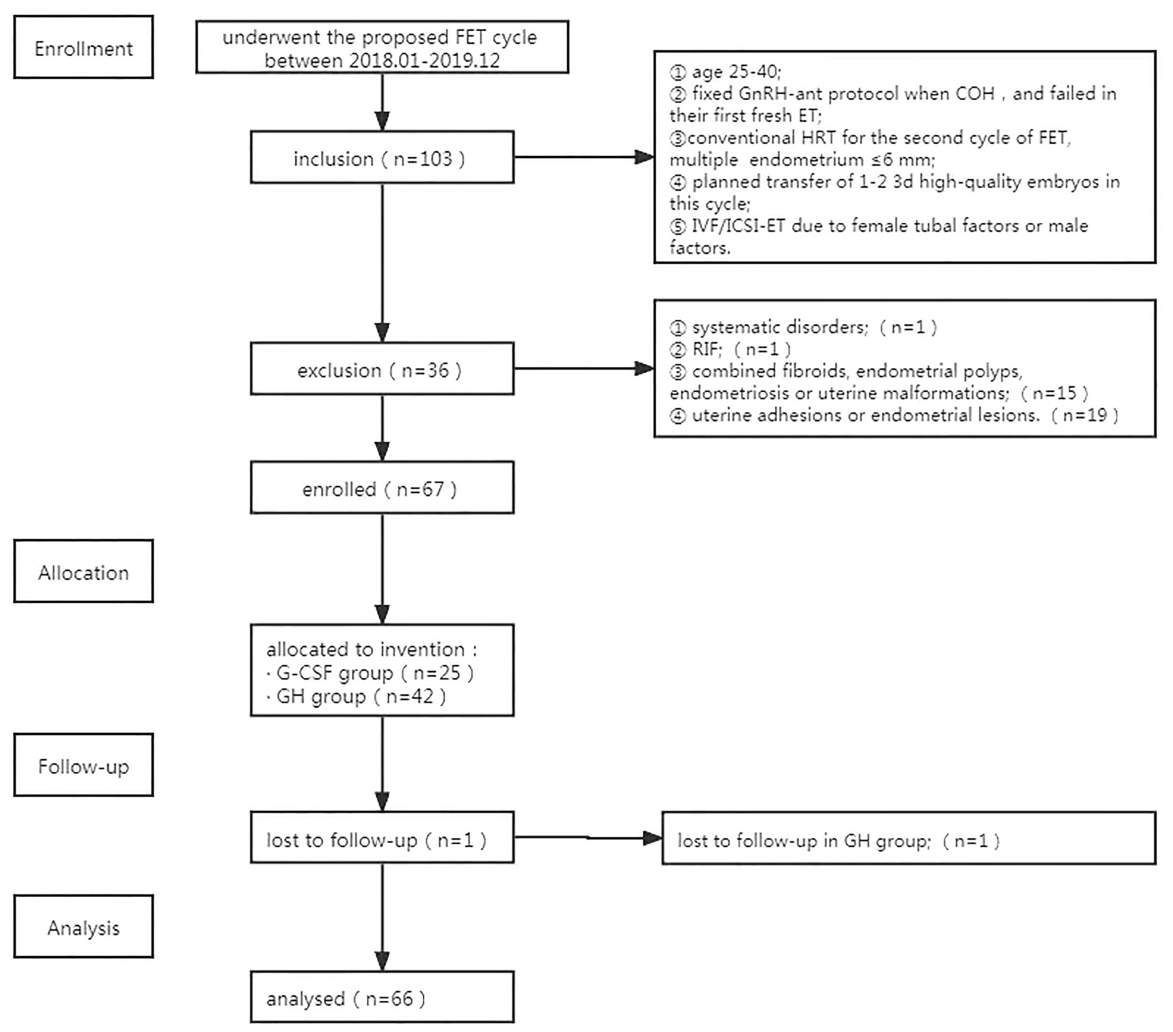
Flowchart of trial enrollment.

A total of 66 patients who met the criteria for nadir discharge were included. G-CSF (n=25) or GH (n=41) was administrated. A flowchart of trial enrolment is shown in **Figure 1**.

##### 1.2.2 Procedure

The controlled ovarian stimulation program was fixed GnRH antagonist protocol. When there were ≥ 3 follicles with diameter ≥ 18mm, hcg5000 ~ 10000IU/ovidrel 250ug was given for trigger, and transvaginal ultrasound-guided puncture was performed 36 hours after trigger. Then IVF/ICSI was selected according to semen quality. Embryo culture was carried out according to the standards of the embryo laboratory of the center. Dosing regimen: all patients routinely underwent urine pregnancy test and transvaginal ultrasonography on day 3 of the menstrual cycle, and received estradiol valerate 2mg bid×4 days + 3mg bid continuously after excluding abnormalities; if the thickness was ≤7mm, the dose was increased to 4mg bid×3d and then monitored again, and if the thickness was still ≤7mm, the dose continued to increase to 5mg bid until the day of P administration. In the perfusion group, granulocyte colony-stimulating factor 1ml was added to the perfusion on the 3rd, 5th, and 7th days of hormone replacement, respectively; in the GH group, GH 5IU/day was added subcutaneously to the start day of hormone replacement until the day of P administration. In both groups, the endometrial condition and serum E2 level were monitored on day 5 (i.e. 48h after the first infusion), day 7 (i.e. 48h after the second infusion), day 9 (i.e. 48h after the third infusion), the day of P administration, and transfer day, respectively, and the medication was adjusted according to ultrasound assessment of endometrial growth; 1-2 3-d embryos of good quality were transferred on the third day after progesterone was given for endometrial transformation. Progesterone gel combined with dydrogesterone was routinely given vaginally for luteal support after the transfer.

##### 1.2.3 Data Collection

① General data of patients, including age, BMI, AMH, basal endocrine, years of infertility, cycle number, number of embryos transferred, and basal endometrial thickness; ② endometrial thickness and morphology on day 5, day 7, day 9, and the day of P administration of the endometrial monitoring cycle (type A is a trilinear type or multi-layered endometrium, showing a hyper-echoic lateral line between the endometrium and myometrium, and two superficial layers of endometrium in close proximity, with a visible hyper-echoic central line; type B is a transitional type, showing isolated echogenicity in the middle and inconspicuous midline echogenicity in the uterine cavity; type C is a homogeneous strongly echogenic type, showing no midline echogenicity in the uterine cavity or a blurred uterine cavity line).

##### 1.2.4 Determination of Pregnancy Outcome

Blood β-hCG > 5 U/L measured 14 days after transplantation was considered as biochemical pregnancy (8), and vaginal ultrasound examination was performed 28-30 days after transplantation, and clinical pregnancy was considered if the gestational sac was visible inside and outside the uterus. Early miscarriage was defined as miscarriage occurring within 12 weeks of gestation. All medications were discontinued if no pregnancy occurred.

#### 1.3 Statistical Methods

SPSS 22.0 software was applied for data analysis. The Shapiro-Wilk test was used for the normal distribution test; the measurement data conforming to the normal distribution were expressed as mean ± standard deviation; a two independent sample t-test was used for comparison between two groups; multiple groups were compared by one-way ANOVA, and *P* < 0.05 was statistically different. Non-normally distributed measures were expressed as median (interquartile spacing); a two independent sample non-parametric test (Mann-Whitney U-test) was used for comparison between two groups, and *P* < 0.05 was statistically different. The statistical data were expressed as rates and compared between groups using the chi-square test or Fisher’s exact probability method, with a statistical difference of *P* < 0.05.

## Results

### 1 Comparison of Basic Data

The comparison of the basic data (age, BMI, AMH, FSH, LH, E_2_, years of infertility, number of embryos transferred, basal endometrial thicknesses, and transformational endometrial thicknesses) of the First Cycle and Second Cycle showed no statistically significant differences (*P* > 0.05), as shown in **Table 1**.

**Table 1:** The comparison of First Cycle and Second Cycle.

	First Cycle (n = 66)	Second Cycle (n = 66)		
	Control Group	G-CSF Group (n = 25)	GH Group (n = 41)	*P*
Age	31.50 ± 4.25	32.68 ± 3.51	31.93 ± 4.23	0.755
BMI (kg/m^2^	22.74 ± 3.18	23.19 ± 3.02	23.80 ± 3.19	1.429
AMH (ng/ml	3.04 ± 2.09	3.22 ± 2.11	2.98 ± 2.62	0.092
FSH (mIU/ml	7.81 ± 3.11	6.75 ± 1.89	7.53 ± 2.37	1.583
LH (mIU/ml	4.85 ± 2.83	4.98 ± 2.04	5.41 ± 3.61	0.456
E_2_ (pg/ml	49.61 ± 39.02	42.91 ± 13.64	60.91 ± 63.02	1.400
Years of infertility (years)	4.30 ± 3.37	5.24 ± 2.47	4.95 ± 2.88	1.073
Number of embryos transferred	1.89 ± 0.31	1.84 ± 0.37	1.95 ± 0.22	1.118
Basal Endometrial thicknesses	4.35 ± 1.13	4.16 ± 0.80	4.13 ± 1.19	0.569
Endometrial thicknesses on the day of P administration	6.59 ± 0.74	6.59 ± 0.61	6.86 ± 0.60	0.103

Continuous variables are expressed as mean ± standard deviation (SD). BMI, body mass index; AMH, anti-Mullerian hormone; FSH, follicle stimulating hormone; LH, luteotropic hormone; E_2_, estradiol; G-CSF, colony cell stimulating factor; GH, Growth Hormone.

### 2 Comparison of Implantation Rate, Biochemical Pregnancy Rate, Clinical Pregnancy Rate, Miscarriage Rate, and Live Birth Rate in the Two Groups

The results showed no statistical difference (*P* > 0.05) in implantation rate, biochemical pregnancy rate, clinical pregnancy rate, miscarriage rate, and live birth rate between the two groups (**Table 2**).

**Table 2:** Comparison of implantation rate, biochemical pregnancy rate, clinical pregnancy rate, miscarriage rate, and live birth rate in the two groups.

	G-CSF Group	GH Group	*P*	OR	95%CI
Implantation Rate	34.8 (16/46)	27.5 (22/80)	0.391	1.406	(0.644, 3.068)
Biochemical Pregnancy Rate	56 (14/25)	48.8 (20/41)	0.569	2.609	(1.005, 6.771)
Clinical Pregnancy Rate	52 (13/25)	46.3 (19/41)	0.655	1.254	(0.463, 3.397)
Miscarriage Rate	23.1 (3/13)	31.6 (6/19)	0.599	0.650	(0.130, 3.260)
Live Birth Rate	40 (10/25)	31.7 (13/41)	0.493	1.436	(0.510, 4.046)

Categorical variables are expressed as percentages (%). G-CSF, colony cell stimulating factor; GH, Growth Hormone.

### 3 Comparison of Endometrial Thickness Between the Two Groups

The endometrial thicknesses of the G-CSF group and GH group were compared on day 5, day 7, day 9, and the day of P administration of the drug, respectively. The results showed that the endometrial thickness in the G-CSF group was thinner than that in the GH group on the 5th of the drug administration, and the difference was statistically significant (P < 0.05), while there was no significant difference in the endometrial thickness on the day 7, day 9, and the day of P administration of administration (**Table 3**).

**Table 3:** The endometrial thicknesses of the G-CSF group and GH group.

	Endometrial thickness		*P*
	G-CSF	GH	
Day 5 of the drug administration	4.83 ± 0.85	5.75 ± 1.27	0.001^*^
Day 7 of the drug administration	5.92 ± 0.86	6.38 ± 0.94	0.053
Day 9 of the drug administration	6.50 ± 0.76	6.57 ± 0.62	0.683
the day of P administration	6.70 ± 0.64	6.87 ± 0.61	0.282

Continuous variables are expressed as mean ± standard deviation (SD). G-CSF, colony cell stimulating factor; GH, Growth Hormone. *P < 0.05, vs GH Group

### 4 Comparison of Endometrial Morphology Between the Two Groups

The endometrial morphology of the G-CSF group and GH group were compared on day 5, day 7, day 9, and the day of P administration, respectively. The results showed that there were differences in the morphology of endometrium between the two groups on day 5. The proportion of endometrial type A morphology in the GH group was significantly higher than that in the G-CSF group on day 5, and the differences were statistically significant (*P* < 0.05), while the type B morphology in the G-CSF group was significantly higher than that in the GH group, and the differences were statistically significant (*P* < 0.05) (**Table 4**).

**Table 4:** Comparison of endometrial morphology between the two groups.

		Type A	Type B	Type C	*P*
Day 5 of the drug administration	G-CSF	28^*^ (7/25)	64^* ^(16/25)	8 (2/25)	0.048
	GH	56.10 (23/41)	34.15 (14/41)	9.76 (4/41)	
Day 7 of the drug administration	G-CSF	76 (19/25)	24 (6/25)	0 (0/25)	0.637
	GH	63.41 (26/41)	34.15 (14/41)	2.44 (1/41)	
Day 9 of the drug administration	G-CSF	76 (19/25)	24 (6/25)	0 (0/25)	0.287
	GH	63.41 (26/41)	36.59 (15/41)	0 (0/41)	
the day of P administration	G-CSF	76 (19/25)	24 (6/25)	0 (0/25)	0.149
	GH	58.54 (24/41)	41.46 (17/41)	0 (0/41)	

Categorical variables are expressed as percentages (%). G-CSF, colony cell stimulating factor; GH, Growth Hormone. *P < 0.05 vs GH Group

### 5 One-Way Logistic Regression Analysis

One-way logistic regression analysis of endometrial thickness and morphology on day 5 of medication in both groups showed that endometrial thickness and morphology on day 5 were not influential factors leading to clinical pregnancy or not (**Table 5**).

**Table 5:** One-way logistic regression analysis of endometrial thickness and morphology.

Elements	
Exposure	Clinical Pregnancy
D5. Endometrial thickness	1.03 (0.61, 1.74) 0.9017
D5. Endometrial morphology	
Type A	1
Type B	1.88 (0.42, 8.44) 0.4127
Type C	0.42 (0.05, 3.84) 0.4396

## Discussion

In China, with the liberalization of the second-child policy, FET cycles are on the rise year by year, and a non-Cochrane systematic study reported that FET cycles have higher clinical pregnancy rates and lower miscarriage rates compared with conventional IVF/ICSI fresh transfer strategies (9). A retrospective study of 513 patients over the age of 35 found that the clinical pregnancy rate and live birth rate were significantly higher in the frozen-thawed embryo transfer cycle than in the fresh embryo transfer cycle (10). This suggests that FET cycles are more beneficial to endometrial development than fresh embryo transfer cycles because of the advantages of reducing the effects of high estrogen on the endometrium during controlled ovarian stimulation (11), preventing ovarian hyperstimulation syndrome, and increasing cumulative pregnancy rates, thus reducing repeated ovulation and egg retrieval in patients (12, 13). Some studies have shown that even in high-quality blastocysts with a score of ≥3BB, the clinical pregnancy rate and the sustained pregnancy rate are only 44% and 41%, respectively (1). Therefore, in addition to good quality embryos, good endometrial receptivity and synchronization between the two are the core factors that influence the outcome of FET cycles. Endometrial receptivity refers to the ability of the endometrium to allow embryo positioning, adhesion, invasion, and induce a series of changes in the endometrial mesenchyme, ultimately allowing embryo implantation. In a normal female menstrual cycle, as follicles gradually grow and mature and estrogen secretion increases, the endometrial thickness will grow to 8-13 mm, providing a suitable environment for embryo implantation, and either too thin or too thick endometrium is not conducive to embryo implantation (14). It is now generally accepted that in ART cycles when the endometrial thickness is 10-14 mm and the morphology is type A, the endometrium is more tolerant, more tolerant to embryo implantation, and has a higher embryo implantation rate. Richter et al. showed that after excluding confounding factors, the pregnancy rate was 53% in those with endometrial thickness of 6-8 mm on HCG day, while the pregnancy rate was 77% in those with endometrial thickness greater than 16 mm (15) and the live birth rate was significantly higher. Goswamy et al. first suggested that inadequate uterine perfusion may be one of the causes of IVF-ET failure (16), and Miwa et al. suggested that it may be due to high blood flow impedance to the terminal branches of the uterine artery (i.e., the uterine spinous artery) (17), which causes ischemic damage to the glandular epithelium of uterine. In 2011, Gleicher et al. first reported that in four patients with thin endometrium who were not sensitive to estrogen and vasodilator drug therapy, the endometrium increased to more than 7 mm after 48 h of intrauterine infusion of G-CSF (4). Embryo transfer resulted in successful pregnancy. Subsequently, Gleicher et al. continued their cohort study in 21 patients and the endometrial thickness increased from 6.4 ± 1.4 mm to 9.3 ± 2.1 mm after intrauterine infusion of G-CSF 6-12 h before trigger (p < 0.001) (18).

Xu and Tehraninejad et al. showed that G-CSF treatment before embryo transfer in patients with thin endometrium thickened the endometrium and facilitated the increase of embryo implantation and pregnancy rate (19, 20). This may be due to the fact that G-CSF stimulates endometrial stem cells or bone marrow stem cells, which promote endometrial growth. These studies are in agreement with our study data showing a progressive trend of increasing endometrial thickness after G-CSF infusion. Furthermore, one study found that in FET cycles, embryos with thin endometrium after GH treatment had significantly improved endometrial thickness on the day of embryo transfer and significantly increased the rate of implantation and clinical pregnancy, and found that under the effect of GH, endometrial cells were able to upregulate the expression of VEGF and ITGB3 and improve endometrial receptivity (7). A meta-analysis showed that GH may promote the expression of vascular endothelial growth factor-1 in the endometrium, inducing endometrial gland hyperplasia, glandular cavity enlargement, and angiogenesis, enrich endometrial stroma, and then significantly increase the thickness of the endometrium, improve the morphology of endometrium, and improve the receptivity of endometrium (21). However, the exact mechanism of GH action on endometrial receptivity remains to be studied in the future. New studies with larger study groups and well-designed RCTs are also needed to clarify whether infertile women with thin endometrium benefit from GH treatment.

In conclusion, a large number of clinical studies have confirmed that G-CSF and GH can improve clinical outcomes in the thin endometrium population; therefore, in this study, we explored which of the two methods is more effective in improving pregnancy outcomes in patients with thin endometrium FET by comparing intrauterine infusion of G-CSF and intramuscular injection of GH, and provided new methods and ideas for clinical treatment. There was no difference between both methods in improving pregnancy outcome, although 48 hours after the first administration of G-CSF, the endometrial thickness was thinner than that in the GH group, and the proportion of Type B endometrial morphology was higher than that in the GH group.

It is conjectured that this might be caused by mechanical damage to the endothelium during the infusion of G-CSF in the uterine cavity. It has been found that G-CSF has the ability to repair wounds and ulcers and has the effect of promoting the proliferation of mucosal cells, thus the addition of G-CSF to the wounds would be beneficial for the increase in thickness (22). During the subsequent administration, it was found that there was no statistical difference in the thickness and morphology of the endothelium between the two groups. The pregnancy rate of all FET patients in our center from 2018 to 2019 is about 50%. In case of failure in the first cycle, the pregnancy rate of thin endometrial patients without treatment remains 30% according to our own data and other previous studies, and it can reach the same level as normal patients if treated with the above two methods (23, 24). Although the results showed no significant differences statistically due to the small sample size, this study has an important clinical value as it showed an increase of 10-20% in the clinical pregnancy rate. This study suggested that the effects of the two methods may be similar, but a large sample of prospective randomized controlled studies is still needed for further exploration.

## Data Availability Statement

The original contributions presented in the study are included in the article/supplementary material. Further inquiries can be directed to the corresponding author.

## Ethics Statement

The study was approved by the ethics committee of the Second Hospital of Hebei Medical University and written informed consent was obtained from all participants.

## Author Contributions

LJ and GH had the conception for the study and designed the study. XX, ZC, and NY did the data analysis. SW, LW, and XHX provided statistical expertise. QL and BS advised on the conduct and coordination of the study. All authors contributed to the interpretation of the results and critical revision of the manuscript for important intellectual content and approved the final version of the manuscript. The corresponding author attests that all listed authors meet authorship criteria and that no others meeting the criteria have been omitted. GH is the guarantor.

## Funding

This study was supported by the Innovation Capability Enhancement Program of Hebei Province (Hebei Clinical Medical Research Center Special Project) (20577710D), the S&T Program of Hebei (20377714D, 21377720D, 21377721D), the Medical Science Research Project of Hebei Province (20211494), the Natural Science Foundation of Hebei Province (Beijing-Tianjin-Hebei Cooperation Special Project) (H2019206707), and the National Key Research and Development Program of China (2018YFC1002104).

## Conflict of Interest

The authors declare that the research was conducted in the absence of any commercial or financial relationships that could be construed as a potential conflict of interest.

## Publisher’s Note

All claims expressed in this article are solely those of the authors and do not necessarily represent those of their affiliated organizations, or those of the publisher, the editors and the reviewers. Any product that may be evaluated in this article, or claim that may be made by its manufacturer, is not guaranteed or endorsed by the publisher.

## References

[1] Al Chami A Saridogan E. Endometrial Polyps and Subfertility. J Obstet Gynaecol India (2017) 67(1):9–14. doi: 10.1007/s13224-016-0929-4 28242961 PMC5306103

[2] Reed ML Said AH Thompson DJ. Large-Volume Vitrification of Human Biopsied and non-Biopsied Blastocysts: A Simple, Robust Technique for Cryopreservation. J Assist Reprod Genet (2015) 32(2):207–14. doi: 10.1007/s10815-014-0395-9 PMC4354176 25464896

[3] Mahajan N Sharma S. The Endometrium in Assisted Reproductive Technology: How Thin is Thin? J Hum Reprod Sci (2016) 9(1):3–8. doi: 10.4103/0974-1208.178632 27110071 PMC4817285

[4] Gleicher N Vidali A Barad DH. Successful Treatment of Unresponsive Thin Endometrium. Fertil Steril (2011) 95(6):2123 e13–7. doi: 10.1016/j.fertnstert.2011.01.143 21324451

[5] Mikolajczyk M Skrzypczak J. Endometrial Receptivity– Can it be Diagnosed and Controlled? And Why Does it Matter? Ginekol Pol (2014) 85(2):149–53. doi: 10.17772/gp/1706 24745162

[6] Hortu I Ozceltik G Sahin C Akman L Erbas O. Granulocyte Colony-Stimulating Factor Prevents Ischemia/Reperfusion-Induced Ovarian Injury in Rats: Evaluation of Histological and Biochemical Parameters. Reprod Sci (2019) 26(10):1389–94. doi: 10.1177/1933719118816839 30497339

[7] Cui N Li AM Luo ZY Zhao ZM Xu YM Zhang J. Effects of Growth Hormone on Pregnancy Rates of Patients With Thin Endometrium. J Endocrinol Invest (2019) 42(1):27–35. doi: 10.1007/s40618-018-0877-1 29671256

[8] Verhaegen J Gallos ID Mello NMV Abdel-Aziz M Takwoingi Y Harb H. Accuracy of Single Progesterone Test to Predict Early Pregnancy Outcome in Women With Pain or Bleeding: Meta-Analysis of Cohort Studies. BMJ (2012) 345:e6077. doi: 10.1136/bmj.e6077 23045257 PMC3460254

[9] Naredi N Singh S Gurmeet P Kumar P Sharma R. Fresh Versus Frozen Embryo Transfer After an In Vitro Fertilization Cycle: Is There a Difference in the Ectopic Pregnancy Rate? Med J Armed Forces India (2021) 77(2):175–80. doi: 10.1016/j.mjafi.2020.03.015 PMC8042501 33867634

[10] Acet F Hortu I Sahin G Goker ENT Tavmergen E. Is Frozen Embryo Transfer Better Than Fresh Embryo Transfer in Women Undergoing Intracytoplasmic Sperm Injection Over the Age of Thirty-Five? A Single Referral Centre Experience. J Obstet Gynaecol (2021) p:1–5. doi: 10.1080/01443615.2021.1882973 33913396

[11] Zapantis G Szmyga MJ Rybak EA Meier UT. Premature Formation of Nucleolar Channel Systems Indicates Advanced Endometrial Maturation Following Controlled Ovarian Hyperstimulation. Hum Reprod (2013) 28(12):3292–300. doi: 10.1093/humrep/det358 PMC3895983 24052503

[12] Roque M. Freeze-All Policy: Is it Time for That? J Assist Reprod Genet (2015) 32(2):171–6. doi: 10.1007/s10815-014-0391-0 PMC4354191 25428436

[13] Roque M Lattes K Serra S Solà I Geber S Carreras R. Fresh Embryo Transfer Versus Frozen Embryo Transfer in In Vitro Fertilization Cycles: A Systematic Review and Meta-Analysis. Fertil Steril (2013) 99(1):156–62. doi: 10.1016/j.fertnstert.2012.09.003 23040524

[14] El-Toukhy T Coomarasamy A Khairy M Sunkara K Seed P Khalaf Y. The Relationship Between Endometrial Thickness and Outcome of Medicated Frozen Embryo Replacement Cycles. Fertil Steril (2008) 89(4):832–9. doi: 10.1016/j.fertnstert.2007.04.031 17681313

[15] Richter KS Bugge KR Bromer JG Levy MJ. Relationship Between Endometrial Thickness and Embryo Implantation, Based on 1,294 Cycles of In Vitro Fertilization With Transfer of Two Blastocyst-Stage Embryos. Fertil Steril (2007) 87(1):53–9. doi: 10.1016/j.fertnstert.2006.05.064 17081537

[16] Goswamy RK Steptoe PC. Doppler Ultrasound Studies of the Uterine Artery in Spontaneous Ovarian Cycles. Hum Reprod (1988) 3(6):721–6. doi: 10.1093/oxfordjournals.humrep.a136772 3220940

[17] Miwa I Tamura H Takasaki A Yamagata Y Shimamura K Sugino N. Pathophysiologic Features of “Thin” Endometrium. Fertil Steril (2009) 91(4):998–1004. doi: 10.1016/j.fertnstert.2008.01.029 18328483

[18] Gleicher N Kim A Michaeli T H-J L Shohat-Tal A Lazzaroni E. A Pilot Cohort Study of Granulocyte Colony-Stimulating Factor in the Treatment of Unresponsive Thin Endometrium Resistant to Standard Therapies. Hum Reprod (2013) 28(1):172–7. doi: 10.1093/humrep/des370 23081869

[19] Xu B Zhang Q Hao J Xu D Li Y. Two Protocols to Treat Thin Endometrium With Granulocyte Colony-Stimulating Factor During Frozen Embryo Transfer Cycles. Reprod BioMed Online (2015) 30(4):349–58. doi: 10.1016/j.rbmo.2014.12.006 25682303

[20] Tehraninejad E Tanha FD Asadi E Kamali K Rezayof E. G-CSF Intrauterine for Thin Endometrium, and Pregnancy Outcome. J Family Reprod Health (2015) 9(3):107–12. PMC4662753 26622308

[21] Momeni M Rahbar MH Kovanci E. A Meta-Analysis of the Relationship Between Endometrial Thickness and Outcome of In Vitro Fertilization Cycles. J Hum Reprod Sci (2011) 4(3):130–7. doi: 10.4103/0974-1208.92287 PMC3276947 22346080

[22] Becher B Tugues S Greter M. GM-CSF: From Growth Factor to Central Mediator of Tissue Inflammation. Immunity (2016) 45(5):963–73. doi: 10.1016/j.immuni.2016.10.026 27851925

[23] Guo Z Chu R Zhang L Yu Q Ma J. Fresh Versus Frozen Embryo Transfer in Women With Thin Endometrium: A Retrospective Cohort Study. Ann Transl Med (2020) 8(21):1435. doi: 10.21037/atm-20-3230 33313180 PMC7723638

[24] Ranisavljevic N Raad J Anahory T Grynberg M Sonigo C. Embryo Transfer Strategy and Therapeutic Options in Infertile Patients With Thin Endometrium: A Systematic Review. J Assist Reprod Genet (2019) 36(11):2217–31. doi: 10.1007/s10815-019-01576-w PMC6885488 31502111

